# Impact of sternotomy and pericardiotomy on cardiopulmonary haemodynamics in a large animal model

**DOI:** 10.1113/EP090919

**Published:** 2023-03-09

**Authors:** Mathilde Emilie Kirk, Victor Tang Merit, Niels Moeslund, Simone Juel Dragsbaek, Jacob Valentin Hansen, Asger Andersen, Mads Dam Lyhne

**Affiliations:** ^1^ Department of Clinical Medicine Aarhus University Aarhus Denmark; ^2^ Department of Cardiology Aarhus University Hospital Aarhus Denmark; ^3^ Department of Cardiac, Lung and Vascular Surgery Aarhus University Hospital Aarhus Denmark; ^4^ Department of Anaesthesiology and Intensive Care Aarhus University Hospital Aarhus Denmark

**Keywords:** gas exchange, porcine, pressure–volume loops, reproducibility, right heart catheterization, rigour, ventricular function

## Abstract

Animal models of cardiovascular disease are often evaluated by invasive instrumentation for phenotyping. As no consensus exists, both open‐ and closed‐chest approaches are used, which might compromise rigour and reproducibility in preclinical research. We aimed to quantify the cardiopulmonary changes induced by sternotomy and pericardiotomy in a large animal model. Seven pigs were anaesthetized, mechanically ventilated and evaluated by right heart catheterization and bi‐ventricular pressure–volume loop recordings at baseline and after sternotomy and pericardiotomy. Data were compared by ANOVA or the Friedmann test where appropriate, with post‐hoc analyses to control for multiple comparisons. Sternotomy and pericardiotomy caused reductions in mean systemic (−12 ± 11 mmHg, *P* = 0.027) and pulmonary pressures (−4 ± 3 mmHg, *P* = 0.006) and airway pressures. Cardiac output decreased non‐significantly (−1329 ± 1762 ml/min, *P* = 0.052). Left ventricular afterload decreased, with an increase in ejection fraction (+9 ± 7%, *P* = 0.027) and coupling. No changes were observed in right ventricular systolic function or arterial blood gases. In conclusion, open‐ versus closed‐chest approaches to invasive cardiovascular phenotyping cause a systematic difference in key haemodynamic variables. Researchers should adopt the most appropriate approach to ensure rigour and reproducibility in preclinical cardiovascular research.

## Abstract (highlights)

- **What is the central question of this study?**
  Invasive cardiovascular instrumentation can occur through closed‐ or open‐chest approaches. To what extent will sternotomy and pericardiotomy affect cardiopulmonary variables?
- **What is the main finding and its importance?**
  Opening of the thorax decreased mean systemic and pulmonary pressures. Left ventricular function improved, but no changes were observed in right ventricular systolic measures. No consensus or recommendation exists regarding instrumentation. Methodological differences risk compromising rigour and reproducibility in preclinical research.

## 1 INTRODUCTION

In preclinical research, the use of animal models mimicking a wide range of cardiovascular disease is increasing (Andersen et al., 2020; Camacho et al., 2016; Houser et al., 2012). Animal models offer the possibility to perform invasive phenotyping of cardiopulmonary haemodynamics, including pressure–volume (*PV*) loops (Burkhoff et al., 2005) that would not be possible or would be unethical to perform in humans.

To perform invasive phenotyping of cardiopulmonary haemodynamics, the heart can be instrumented based on an open‐ or closed‐chest approach, but cardiopulmonary haemodynamics will be altered by sternotomy and pericardiotomy (Edde & Miller, 1981; Waller et al., 1981). There are no guidelines or consensus on which approach to use, as evident by the different approaches adoped in the literature on different cardiovascular models (Berry et al., 2004; Boulate et al., 2019; Chua et al., 2013; Gold et al., 2020; Kerbaul et al., 2011; Lyhne, Schultz, Kramer et al., 2021; Moeslund et al., 2023; Vammen et al., 2021).

The different instrumentation approaches for invasive phenotyping might impair comparison between studies, and only few data on the impact of such methodological differences on cardiopulmonary haemodynamics have been published. To improve rigour and reproducibility in preclinical research using invasive phenotyping of cardiopulmonary haemodynamics, more knowledge on the impact of sternotomy and pericardiotomy is needed.

In this study, we aimed to quantify the effects of sternotomy and pericardiotomy on cardiopulmonary haemodynamics in a large animal model.

## 2 METHODS

### 2.1 Ethical approval

This study was approved by the Danish Animal Research Inspectorate (license number: 2018‐15‐0201‐01521) and adhered to the ARRIVE guidelines on animal research (Sert et al., 2020). Handling and transportation of animals were in accordance with Denmark's Animal Welfare Act 2013. The investigators understand the ethical principles under which the journal operates, and the work complies with the animal ethics checklist outlined by the journal. This study was conducted in continuation of another study, and hereby respecting the 3R principles of reduction, replacement and refinement. In addition, each animal served as its own control to reduce the necessary number of animals.

### 2.2 Animals and handling

We used Danish female slaughter pigs (crossbred of Danish Landrace/Yorkshire and Duroc) weighing 75 ± 3 kg, corresponding to 5–6 months of age. Pigs followed the specific pathogen‐free programme, and no genetic modification was used. Animals were acclimated for 2 weeks before protocol and were observed daily by qualified personnel. Pigs received a conventional diet twice daily, except when fasting before the procedure. Pigs were housed in pens with a concrete floor and straw bedding either in pairs or alone with the possibility of snout contact and were let out daily for free physical activity. The light–dark cycle was 12 h–12 h.

### 2.3 Study design

**FIGURE 1:**
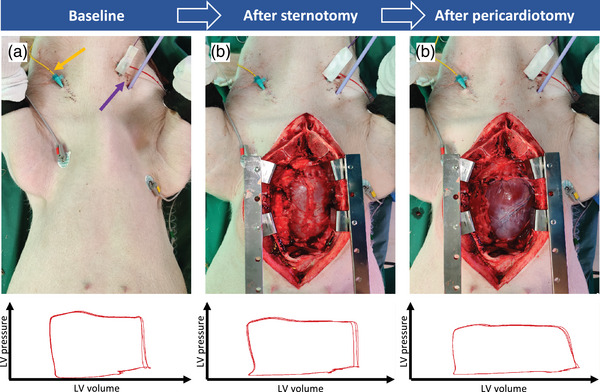
Study design. Cardiopulmonary function was evaluated at baseline with closed chest (a), with open chest after sternotomy (b) and with open pericardium after pericardiotomy (c). In (a), the yellow arrow marks the entrance of a Swan‐Ganz catheter for right heart catheterization, and the purple arrow marks the entrance point of pressure–volume loop catheters for both ventricles. Below are representative left ventricular (LV) pressure–volume loops shown for each consecutive step.

This was an experimental study of repeated measurements. Cardiopulmonary haemodynamics were evaluated and compared at three consecutive time points: (1) at baseline with closed chest; (2) with open chest after sternotomy; and (3) with open chest and open pericardium after pericardiotomy (Figure 1). Each animal served as an experimental unit. Randomization was not possible in this design. Cardiopulmonary haemodynamics were evaluated at each time point with bi‐ventricular *PV* loop recordings, right heart catheterization (RHC), invasive blood pressure measurements, ventilator variables including end‐tidal CO_2_ (EtCO_2_), and arterial and mixed venous blood gases. The animals were killed by exsanguination under deep general anaesthesia at the end of the protocol.

The vascular accesses and closed‐chest catheter instrumentation lasted 2 h before baseline evaluation. Sternotomy was performed carefully over ∼10 min and pericardiotomy over 10 s. Each measurement was acquired ≥5 min after sternotomy and pericardiotomy, respectively, to allow the cardiopulmonary haemodynamics to stabilize, and each measurement lasted ∼10 min.

This was a secondary study performed in continuation of another study. The original study included a 30‐day follow‐up protocol after pulmonary emboli. All pigs underwent the intervention with pulmonary emboli.

We included animals that completed the study protocol. Exclusion criteria were established a priori and included unsuccessful surgical procedures, significant blood loss or inability to obtain reliable *PV* data. Three pigs were excluded [one owing to unreliable *PV* loop acquisition, one owing to significant pericardial perforation during thoracotomy, and one owing to haemodynamic instability during inferior vena cava (IVC) occlusions]. Data points from load‐independent *PV* measurements of the left ventricle (LV) were excluded from one pig, and pulmonary capillary wedge pressure (PCWP) data were excluded from two pigs, both owing to unreliable data.

### 2.4 Anaesthesia and ventilation

Animals were pre‐anaesthetized with an intramuscular injection of Zoletil Mix 0.1 mL/kg (tiletamine 25 mg/mL, zolazepam 25 mg/mL, butorphanol 10 mg/mL, ketaminol 100 mg/mL and xylazine 20 mg/mL; Virbac, Denmark). General anaesthesia was maintained by i.v. infusion of propofol 4.0 mg/kg/h (10 mg/mL; Fresenius Kabi, Denmark) and fentanyl 12.5 μg/kg/h (50 μg/mL; B. Braun Medical, Denmark). No neuromuscular blockers were used, and absence of reflexes was checked regularly to ensure sufficient anaesthetic depth. Animals were intubated and ventilated with a respiratory rate of 14 ± 1 breaths/min, tidal volume of 500 mL and positive end‐expiratory pressure of 5 cmH_2_O. We observed no changes in temperature during procedures.

### 2.5 Haemodynamic measurements

Invasive arterial blood pressure was measured through a 6 French sheath (Terumo Radifocus Introducer II, Japan) in the left femoral artery. Heart rate and mean arterial pressure (MAP) were recorded.

Pressure transducers were reset before instrumentation at the level of the animal's heart in room air. Right heart catheterization with a Swan‐Ganz catheter (7.5 French, CCOmbo; Edwards Lifescience, USA) was performed through an 8 French sheath in the right external jugular vein and positioned with the tip in the main pulmonary artery, guided by fluoroscopy and the pressure curve. In this position, pulmonary arterial pressure (PAP) was measured from the tip, and central venous pressure (CVP) was measured from the proximal pressure transducer located in the right atrium, without movement of the catheter. To obtain PCWP, the catheter balloon was inflated and the catheter advanced until the pressure curve showed a wedge pressure. After measurement of PCWP, the catheter was returned to its original position. Cardiac output (CO) was measured by thermodilution as an average of three injections of 10 mL 5% glucose of 5°C within a 10% margin, and stroke volume (SV) was calculated. Pulmonary vascular resistance (PVR) was calculated using LV end‐diastolic pressure (EDP) [PVR = (mPAP − LV EDP)/CO, where mPAP is the mean pulmonary arterial pressure]. Systemic vascular resistance (SVR) was calculated as (MAP − CVP)/CO. The PCWP was omitted from the PVR calculation owing to unreliable measurements in two animals.

### 2.6 Pressure–volume loop instrumentation and measurements

The instrumentation has been described in detail previously (Lyhne, Schultz, Dragsbaek et al., 2021, Lyhne, Schultz, Kramer et al., 2021). All instrumentations were established using a closed‐chest, minimally invasive approach guided by ultrasound and fluoroscopy. The *PV* catheters (Transonic Scisence, London, ON, Canada) were advanced to the LV through an 8 French sheath in the left carotid artery and to the right ventricle (RV) through a 16 French sheath (Check‐Flo Performer, Cook Medical, USA) in the left external jugular vein. Catheters were maintained in this position during the following sternotomy, although other models of open‐chest research use an apical approach to the ventricles. By not changing the *PV* instrumentation approach, our observed changes would be attributable to sternotomy and pericardiotomy and not to the instrumentation methodology, myocardial damage or bleeding that could otherwise happen by the apical approach. The *PV* data were recorded through admittance control units (ADV500; Transonic Scisense) and PowerLab 8/35 (ADInstruments, Oxford, UK; RRID: SCR_018833). An IVC balloon (PTS‐X; Metec, Denmark) was inserted through a 10 French sheath in the femoral vein. Inflation of the IVC balloon gradually reduced preload and allowed recording of load‐independent *PV* data. The balloon was immediately deflated, and occlusion was repeated when haemodynamics were stable.

Load‐dependent data were recorded during ongoing respiration, and an average of three respiratory cycles was used in the analysis. Data included end‐systolic and end‐diastolic pressures (ESP and EDP, respectively) and volumes (ESV and EDV, respectively), ejection fraction (EF) and t (τ Weiss method). Stroke work (SW) represents the area of the *PV* loop and was provided automatically by the software. Load‐independent data were recorded during transient end‐expiratory breath hold, and an average of three IVC occlusions was used to determine end‐systolic elastance (Ees) and end‐diastolic elastance (Eed) (Burkhoff et al., 2005). Arterial elastance (Ea) recorded during breath hold was used to calculate ventriculo‐arterial coupling (Ees/Ea). Data were analysed in LabChart, with the observer blinded to the time points. Recorded *PV* data files were blinded by allocating a random letter for each animal and a random three‐digit number for each time point. Blinding and unblinding was performed by one researcher and analysis by another.

### 2.7 Biochemical analysis

Arterial and mixed venous blood gases were sampled simultaneously and analysed immediately (ABL90 Flex Plus; Radiometer Medical, Denmark). Physiological dead space was calculated (Zwissler et al., 1995).

### 2.8 Statistics

The sample size of the original study was calculated a priori as *n* = 6. We performed four pilot studies, and the present secondary study was initiated on all 10 pigs to increase power. Variables were tested for normal distribution using the Shapiro–Wilks test and QQ‐plots. Data are presented as the mean ± SD if normally distributed and the median [IQR] if not. The three time points were compared using one‐way ANOVA of repeated measurements or the Friedmann test where appropriate. If values of one time point were not normally distributed, a non‐parametric test was used to compare the time points. Post‐hoc analyses were performed using Tukey's or Dunn's multiple comparisons test where appropriate. Statistical analyses were performed on the absolute values, but data are presented as differences from the closed‐chest state according to the purpose of the study. A *P*‐value < 0.05 was considered statistically significant. GraphPad Prism v.9.4.0 (GraphPad Software, San Diego, CA, USA; RRID: SCR_002798) was used for statistical analyses.

## 3 RESULTS

Ten pigs were enrolled, and three animals were excluded. Accordingly, seven animals were included in the final analyses.

Baseline data of absolute values in closed‐chest conditions are provided in Table 1. We observed generally normal values but relatively high CO. Absolute values for all cardiopulmonary variables at baseline with closed chest, after sternotomy and after pericardiotomy can be found in the Supporting Information (Table S1).

**TABLE 1:** Absolute values of haemodynamics, ventilation and ventricular function at baseline in closed‐chest conditions.

Haemodynamics			
Heart rate, /min	61 ± 17	CVP, mmHg	3 ± 2
MAP, mmHg	75 ± 10	PCWP, mmHg (*n* = 5)	8 ± 4
CO, mL/min	6600 [5300–8500]	PVR, dyn/s/cm^5^	144 ± 40
SV, mL	117 ± 18	SVR, dyn/s/cm^5^	900 ± 304
mPAP, mmHg	20 ± 5	PVR/SVR	0.180 ± 0.095
**Ventilation**			
Arterial pH	7.45 ± 0.03	Mixed venous O_2_ content, mL/dL	7.54 ± 2.53
$P_{{\mathrm{aCO}}_{2}}$, kPa	6.31 ± 0.47	Arteriovenous O_2_ difference, mL/dL	4.49 ± 1.03
$P_{{\mathrm{aO}}_{2}}$, kPa	10.80 [8.66–19.40]	Physiological dead space, mL	2 ± 21
$P_{{\mathrm{vCO}}_{2}}$, kPa	7.24 ± 0.40	EtCO_2_, kPa	6.3 ± 0.6
$P_{{\mathrm{vO}}_{2}}$, kPa	5.30 ± 1.07	*P*_peak_, cmH_2_O	18 ± 3
Mixed venous saturation, %	59.8 ± 12.7	*P*_mean_, cmH_2_O	10 [8–11]
**Ventricular function**			
**Variable**	**LV**	**RV**	
ESP, mmHg	89 ± 11	32 ± 4	
EDP, mmHg	9 [8–9]	5 ± 2	
ESV, mL	53 ± 21	28 ± 19	
EDV, mL	172 [157–225]	149 ± 57	
EF, %	72 [55–74]	75 ± 16	
Ea, mmHg/mL	0.659 [0.573–1.625]	0.286 ± 0.132	
SW, mmHg*mL	8876 ± 3644	1863 [1306–3348]	
τ, ms	34 ± 4	39 [36–63]	
d*P*/d*t*_min_, mmHg/s	−1940 ± 502	−513 ± 70	
Ees, mmHg/mL	0.378 [0.251–0.677]^*^	0.548 ± 0.339	
Eed, mmHg/mL	0.079 [0.052–0.125]^*^	0.063 [0.040–0.073]	
Ees/Ea	0.523 ± 0.260^*^	2.359 ± 1.208^*^	

*Note*: Data are presented as the mean ± SD or median [interquartile range] where appropriate. *n* = 7 for all except ^*^ *n* = 6.

Abbreviations: CO, cardiac output; CVP, central venous pressure; Ea, arterial elastance; EDP, end‐diastolic pressure; EDV, end‐diastolic volume; Ees, end‐systolic elastance; Eed, end‐diastolic elastance; EF, ejection fraction; ESP, end‐systolic pressure; ESV, end‐systolic volume; EtCO_2_, end‐tidal CO_2_; d*P*/d*t*, derivative of pressure over time; LV, left ventricular; MAP, mean arterial pressure; mPAP, mean pulmonary arterial pressure; *P*_mean_, mean airway pressure; *P*_peak_, peak airway pressure; PCWP, pulmonary capillary wedge pressure; $P_{\mathrm{aC}O_{2}}$, partial pressure of arterial CO_2_; $P_{aO_{2}}$, partial pressure of arterial O_2_; $P_{\mathrm{vC}O_{2}}$, partial pressure of mixed venous CO_2_; $P_{vO_{2}}$, partial pressure of mixed venous O_2_; PVR, pulmonary vascular resistance; RV, right ventricular; SV, stroke volume; SVR, systemic vascular resistance; SW, stroke work.

**FIGURE 2:**
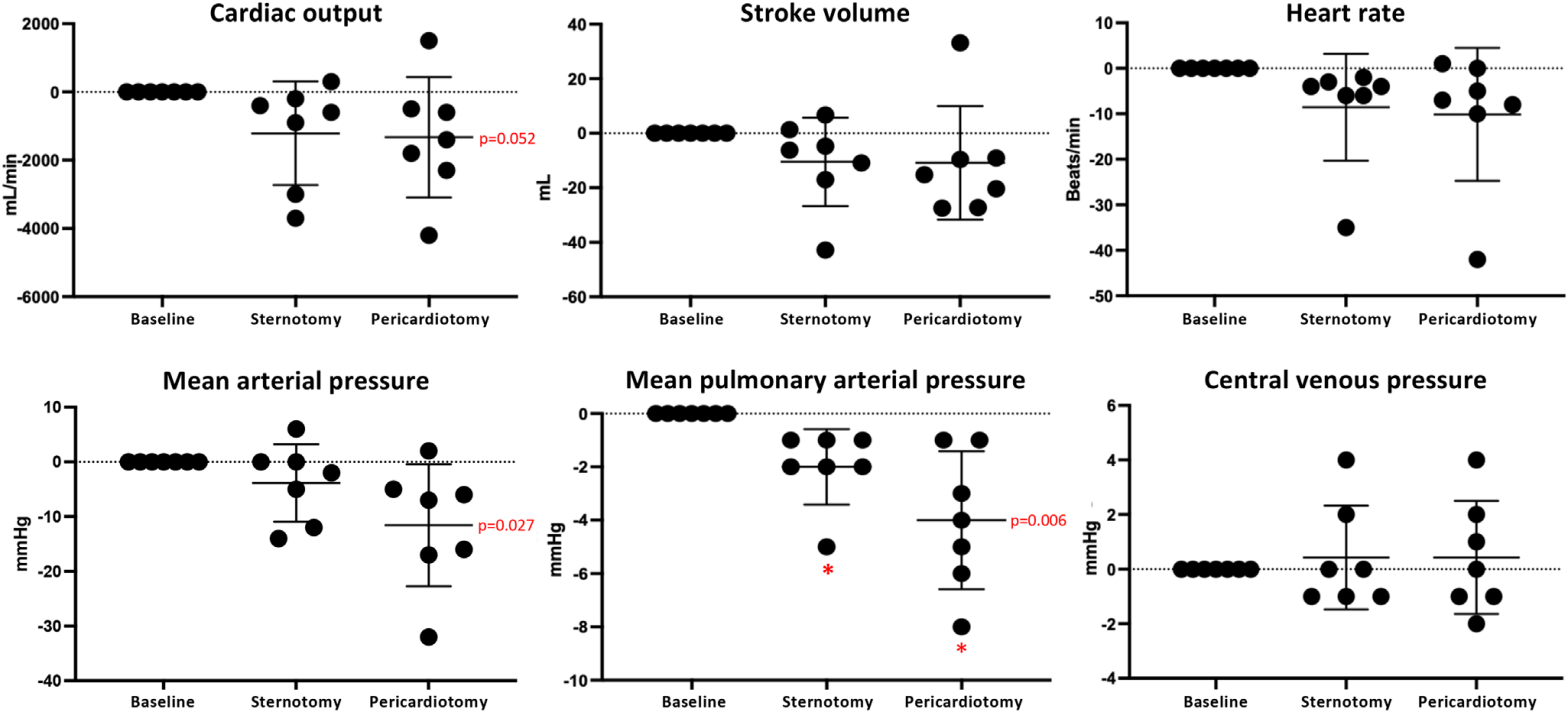
Haemodynamic changes from sternotomy and pericardiotomy. Data are shown as absolute changes from baseline (closed chest) and presented as the mean ± SD. *P*‐values represent the overall ANOVAs. ^*^ *P* < 0.05 versus baseline in post‐hoc analysis.

Compared with closed‐chest conditions, we observed a decrease in mPAP (−4 ± 3 mmHg, *P* = 0.006) and MAP (−12 ± 11 mmHg, *P* = 0.027) after sternotomy and pericardiotomy (Figure 2). A statistically non‐significant change in CO (−1329 ± 1762 mL/min, *P* = 0.052) induced by sternotomy and pericardiotomy was driven by changes in both heart rate and SV (Figure 2). No changes were observed for CVP, PCWP, PVR or SVR (Figure 2; Table 2).

**TABLE 2:** Absolute changes in haemodynamics after sternotomy and pericardiotomy.

**Variable**	**Change after sternotomy**	***P*‐value (sternotomy vs. baseline)**	**Change after pericardiotomy**	***P*‐value (pericardiotomy vs. baseline)**	***P*‐value (pericardiotomy vs. sternotomy)**	***P*‐value (overall ANOVA)**
**Haemodynamics**						
PCWP, mmHg^*^	1 ± 2	0.677	0 ± 1	0.938	0.905	0.670
PVR, dyn/s/cm^5^	−4 ± 40	0.964	−23 ± 44	0.392	0.250	0.263
SVR, dyn/s/cm^5^	136 ± 190	0.220	54 ± 141	0.599	0.193	0.117
PVR/SVR	−0.026 ± 0.020	0.035	−0.038 ± 0.044	0.136	0.640	0.065
**Ventilation**						
Arterial pH	0.005 ± 0.011	0.524	0.004 ± 0.014	0.795	0.364	0.303
$P_{\mathrm{aC}O_{2}}$, kPa	0.10 ± 0.26	0.594	−0.06 ± 0.23	0.781	0.365	0.296
$P_{aO_{2}}$, kPa	−0.49 [−0.70 to 0.30]	>0.999	0.30 [−0.72 to 0.79]	>0.999	>0.999	0.964

*Note*: Absolute differences in cardiopulmonary variables at baseline with closed chest and closed pericardium from conditions after sternotomy with open chest but closed pericardium and after pericardiotomy with open chest and open pericardium. Data are presented as the mean ± SD or median [interquartile range] where appropriate. *n* = 7 for all except ^*^ *n* = 5.

Abbreviations: $P_{\mathrm{aC}O_{2}}$, partial pressure of arterial CO_2_; $P_{aO_{2}}$, partial pressure of arterial O_2_.; PCWP, pulmonary capillary wedge pressure; PVR, pulmonary vascular resistance; SVR, systemic vascular resistance.

**FIGURE 3:**
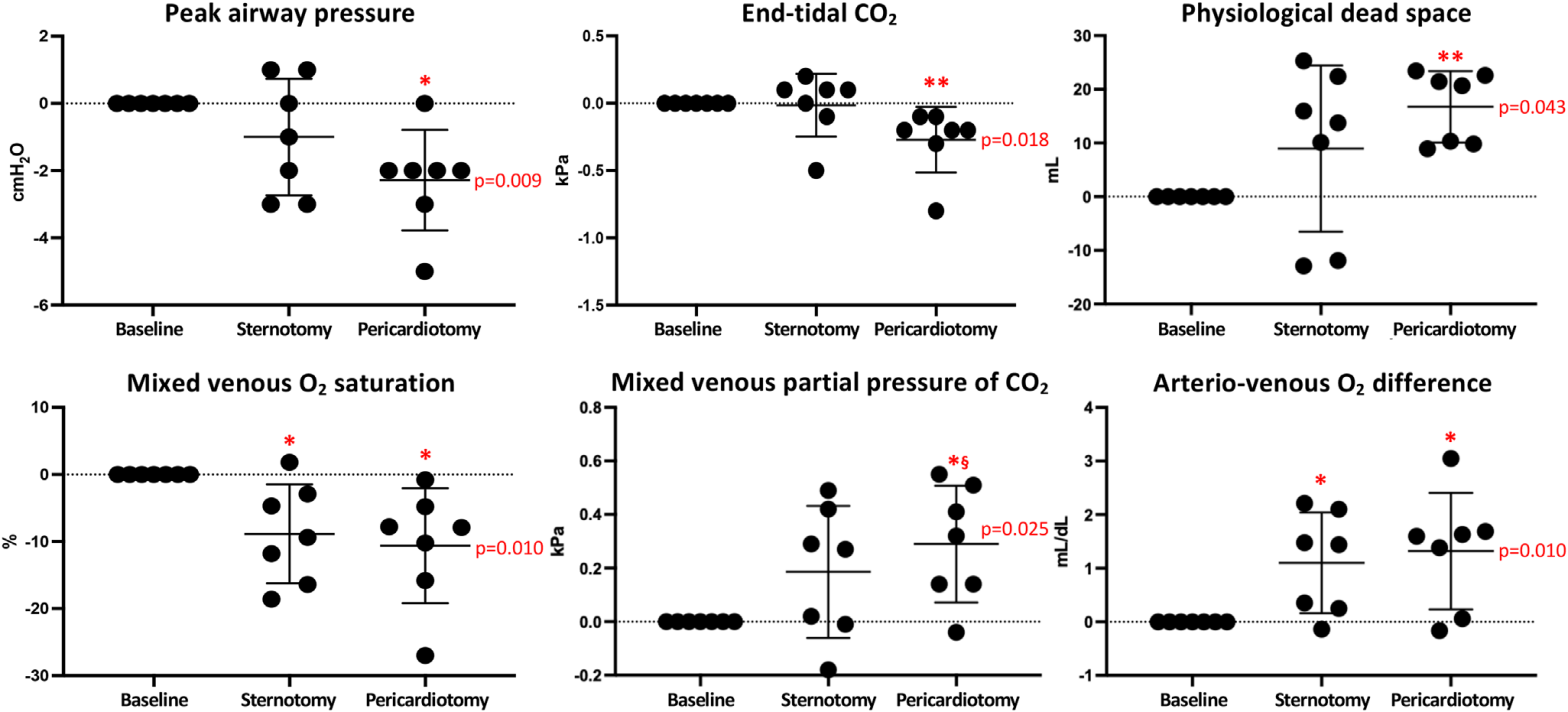
Ventilatory changes from sternotomy and pericardiotomy. Data are shown as absolute changes from baseline (closed chest) and presented as the mean ± SD. *P*‐values represent the overall ANOVAs. ^*^ *P* < 0.05 versus baseline, ^**^ *P* < 0.01 versus baseline in post‐hoc analysis; ^§^ *P* < 0.05 versus sternotomy in post‐hoc analysis.

Regarding ventilatory function, sternotomy and pericardiotomy caused a decrease in airway pressures (−2 ± 1 cmH_2_O, *P* = 0.009) and EtCO_2_ (−0.2 [−0.3; −0.1] kPa, *P* = 0.018; Figure 3). Arterial blood gases were not affected by sternotomy and pericardiotomy, but mixed venous blood gases showed decreased venous saturation (−10.6 ± 8.6%, *P* = 0.010; Figure 3) and venous O_2_ content (−1.53 ± 1.35 mL/dL, *P* = 0.017) and increased partial pressure of CO_2_ (0.29 ± 0.22 kPa, *P* = 0.025; Figure 3). For further details, see Table 2 and the Supporting Information (Tables S1 and S2).

**FIGURE 4:**
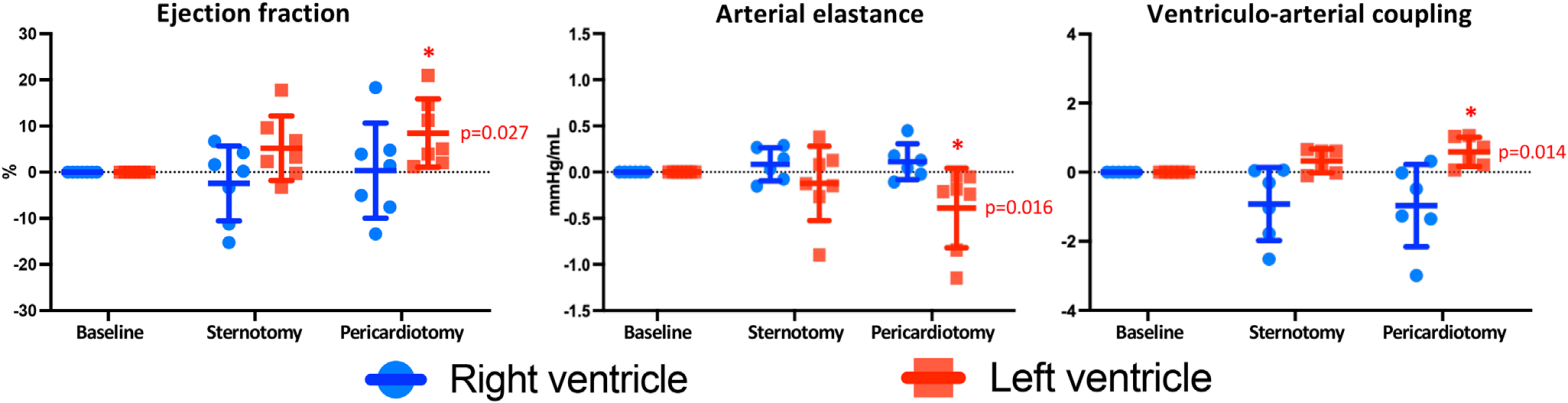
Changes in ventricular function from sternotomy and pericardiotomy. Left ventricular data are shown in red and right ventricular data in blue. Data are shown as absolute changes from baseline (closed chest) and presented as the mean ± SD. *P*‐values represent the overall ANOVAs. ^*^ *P* < 0.05 versus baseline in post‐hoc analysis.

Compared with the baseline closed‐chest conditions, sternotomy and pericardiotomy caused few changes in RV and LV function. The LV Ea decreased (−0.21 [−0.85; −0.05] mmHg/mL, *P* = 0.016), with a concomitant increase in LV coupling (0.587 ± 0.424, *P* = 0.014) and LV EF (9 ± 7%, *P* = 0.027; Figure 4). We observed no changes in RV systolic function (Figure 4). For further details on changes in LV and RV function, see Table 3 and the Supporting Information (Tables S1 and S2).

**TABLE 3:** Absolute changes in ventricular function after sternotomy and pericardiotomy.

**Variable**	**Change after sternotomy**	***P*‐value (sternotomy vs. baseline)**	**Change after pericardiotomy**	***P*‐value (pericardiotomy vs. baseline)**	***P*‐value (pericardiotomy vs. sternotomy)**	***P*‐value (overall ANOVA)**
**Left ventricular function**						
LV ESP, mmHg	−5 ± 10	0.446	−9 ± 9.0	0.099	0.258	0.076
LV EDP, mmHg	0 ± 1	>0.999	0 ± 2	>0.999	>0.999	0.768
LV ESV, mL	−10 ± 9	0.062	−7 ± 14	0.461	0.820	0.145
LV EDV, mL	7 [−23 to 15]	>0.999	11 [−5 to 76]	0.544	0.855	0.486
LV SW, mmHg*mL	499 ± 1650	0.629	1419 ± 1890	0.155	0.420	0.116
LV τ, ms	2 [0–7]	0.075	4 [2–9]	0.011	0.005	0.005
LV d*P*/d*t*_min_, mmHg/s	291 ± 504	0.516	418 ± 468	0.157	0.056	0.121
LV Ees, mmHg/mL^*^	0.145 [0.049–0.592]	0.063	0.157 [−0.028 to 0.359]	0.130	>0.999	0.052
LV Eed, mmHg/mL^*^	0.003 [−0.049 to 0.127]	>0.999	−0.039 [−0.096 to −0.009]	0.130	0.063	0.052
**Right ventricular function**						
RV ESP, mmHg	−0 ± 2	0.990	0 ± 3	0.982	0.918	0.909
RV EDP, mmHg	2 ± 2	0.082	2 ± 2	0.120	0.994	0.034
RV ESV, mL	3 ± 7	0.567	4 ± 9	0.602	0.844	0.370
RV EDV, mL	25 ± 101	0.799	26 ± 70	0.618	0.999	0.548
RV SW, mmHg*mL	−21 ± 1306	>0.999	−78 ± 940	0.326	>0.999	0.305
RV τ, ms	17 ± 18	0.049	18 ± 11	0.049	>0.999	0.021
RV d*P*/d*t*_min_, mmHg/s	40 ± 81	0.444	58 ± 53	0.062	0.590	0.106
RV Ees, mmHg/mL	−0.061 [−0.187 to 0.001]	0.332	−0.057 [−0.129 to 0.005]	0.334	0.786	0.295
RV Eed, mmHg/mL	−0.028 [−0.039 to −0.003]	0.544	−0.045 [−0.055 to 0.083]	0.184	>0.999	0.192

*Note*: Absolute differences in ventricular function at baseline with closed chest and closed pericardium from conditions after sternotomy with open chest but closed pericardium and after pericardiotomy with open chest and open pericardium. Data are presented as the mean ± SD or median [interquartile range] where appropriate. *n* = 7 for all except ^*^ *n* = 6.

Abbreviations: EDP, end‐diastolic pressure; EDV, end‐diastolic volume; Eed, end‐diastolic elastance; Ees, end‐systolic elastance; ESP, end‐systolic pressure; ESV, end‐systolic volume; LV, left ventricular; RV, right ventricular; SW, stroke work.

## 4 DISCUSSION

In the present study, we demonstrated and quantified systematic differences in cardiopulmonary haemodynamics between closed‐chest conditions and after sternotomy and sternotomy plus pericardiotomy. We observed that pulmonary and arterial pressures decreased after the interventions, and we observed a clinically and scientifically relevant reduction in CO. The RV function was generally unaffected, whereas LV EF and coupling increased.

Our findings are important for the rigour and reproducibility in preclinical cardiovascular research, in concordance with previous studies on methodological effects on haemodynamic measurements (Lyhne et al., 2022). Closed‐chest instrumentation of the heart is possible (Lyhne, Schultz, Dragsbaek et al., 2021) and would most often mimic the clinical scenario most closely, whereas for other models, such as heart transplantation or open‐heart surgery, an open‐chest approach would be of relevance (Moeslund et al., 2023). Other instrumentation approaches, including flow probe placement, require reclosing of the thorax to mimic closed‐chest physiology (Mitchell et al., 2005; Pinsky et al., 1983). However, exactly recreating negative intracavity pressures and chest wall stability is impossible, and incomplete haemostasis or oedema in the surgical field changes intrathoracic mechanical circumstances compared with pre‐sternotomy. The RV longitudinal function is also impaired by sternotomy and even worsens after chest closure (Korshin et al., 2019). Collectively, reclosing the thorax after instrumentation would, presumably, alter haemodynamics towards the initial closed‐chest conditions, but cannot recreate pre‐sternotomy physiology.

The physiological effects of cardiac constraint by the pericardium, lungs and chest wall and how the removal of an external constraint will reduce the impact of any intervention of interest have been discussed for decades (Belenkie et al., 2004; Butler, 1983; Lloyd, 1982; Pinsky, 2020; Pinsky et al., 1983), supporting our aim of quantifying the changes on modern cardiovascular equipment in order to improve rigorous reporting. Furthermore, we emphasize the importance of researchers choosing a clinically relevant animal model (open vs. closed chest) mimicking their field of interest, but we cannot provide a single best model for specific cardiac disease paradigms. Instead, we aim to draw the attention of researchers to the magnitude of differences in the present study and to the risk of falsely negative studies in models lacking the external cardiac constraint (Belenkie et al., 2004). It has previously been determined how cardiac function is augmented by changes in intrathoracic pressure (Pinsky et al., 1985), but open‐chest approaches do change the circumstances and cardiorespiratory interactions completely.

Some guidelines on methodological approaches in preclinical cardiovascular research have been published (Lindsey et al., 2018; Wenceslau et al., 2021). No recommendation or consensus on the intracardiac approach for invasive phenotyping exists, despite its potential impact on rigour in animal research.

### 4.1 Systemic and pulmonary haemodynamics

We noticed a stepwise decrease in mPAP. Sternotomy abolishes the restrictive forces of the chest wall, and positive pressure ventilation will cause lung expansion. According to the law of Laplace, airway pressure will diminish, as confirmed by our findings and human data (Arslan et al., 2019). Airway pressure is the driving force for lung perfusion in West's lung zones 1 and 2 (Kreit, 2022; West et al., 1964). When airway pressure drops, a larger proportion of the lung will be zone 3; that is, pulmonary vascular recruitment will occur, with a larger cross‐sectional area for perfusion. The larger pulmonary vascular bed can explain, at least in part, our observations of mPAP reduction.

Cardiac output showed a statistical trend towards a decrease (*P* = 0.052) and was affected by both heart rate and SV changes and in concordance with the observed reduction in EtCO_2_ and mixed venous saturation. The magnitude of CO reduction was, however, of scientific relevance (>1 L/min).

Cardiac output is dependent primarily on venous return in conditions of fixed lung volume (closed chest), but ventricular function is depressed, causing reduced CO when open‐chest conditions alter lung volumes, as discussed elsewhere (Butler, 1983). Our results are comparable to human data where sternotomy caused a decrease in CO and SV, with no effects on other key variables, such as CVP or PCWP (Edde & Miller, 1981; Waller et al., 1981). Pinsky et al. (1983) performed an intervention opposite to ours by applying chest binders to the animals and observed haemodynamic changes opposite to ours (i.e., increasing SV and CO). They explained the increase by an improved pressure gradient from inside to outside the thoracic cavity, supporting LV output (Pinsky et al., 1983). In our study, sternotomy and pericardiotomy abolish the transthoracic ejection pressure gradient, lowering LV output. Other studies, both clinical and preclinical, did not find changes in MAP or CO but do agree on the lack of changes in CVP and PCWP, as in our study (Angelini et al., 1990; Blasi et al., 2007; Korshin et al., 2019). The cause of the differences might be interspecies dependent or the lack of differentiation between sternotomy and pericardiotomy (Blasi et al., 2007), and it emphasizes the importance of rigorous reporting of the approach used to perform invasive haemodynamic evaluation.

### 4.2 Ventricular functions

Pericardiotomy trended to cause LV dilatation, as shown significantly previously (Angelini et al., 1990) and decreased ESP, partly explained by the law of Laplace and the lower ejection pressure gradient (see above). The reduction in LV ESP compared with the unchanged RV ESP reduced the transseptal pressure gradient in systole (Pinsky, 2020). Changes in transseptal pressure gradient might normally cause septal shift and changes in ventricular volumes. That type of ventricular interdependence is abolished in our case of pericardiotomy, confirmed by unaltered cardiac volumes.

Despite the lowered ejection pressure, a concomitant reduction in LV afterload and increased intrinsic contractility improved left ventriculo‐arterial coupling and LV EF. Belenkie et al. (2004) showed significantly increased LV size but also SV upon pericardiotomy in a model of acute RV pressure overload. Releasing the constraint from the pericardium improved the transseptal pressure gradient and the LV filling and output. This is different to our study without pathological interventricular circumstances and impaired LV filling before pericardiotomy. The ventricular effect of pericardiotomy thereby depends on the pre‐interventional situation, which confirms previous findings (Pinsky, 2020). Likewise, interventricular dependence has been shown to vary with intrathoracic pressure (Mitchell et al., 2005), but sternotomy and pericardiotomy change those circumstances.

Contrary to the LV, we did not observe alterations from pericardiotomy on RV systolic function, comparable to others (Belenkie et al., 2004). Clinical studies in humans have shown similar RV function based on echocardiography (Labus et al., 2021). Echocardiographic measures of ventricular function are often load dependent, and we have now confirmed these findings using invasive, state‐of‐the‐art *PV* measurements including load‐independent variables of ventricular function.

In diastole, we observed alterations in τ and d*P*/d*t*_min_ in a similar direction towards diastolic impairment. However, both the rate of pressure decay and the τ Weiss are known to be very dependent on respiration and load (Ogilvie et al., 2020), and loading conditions change substantially with sternotomy and pericardiotomy. Any deformation of the lungs and chest wall affects the elastic load on ventricular diastolic filling (Lloyd, 1982), which we removed by our intervention. In fact, the end‐diastolic *PV* relationship (Eed) trended to change in the opposite direction. In both ventricles, we observed the largest alterations of Eed from sternotomy to pericardiotomy, supporting the previous finding that diastolic interdependence requires an intact pericardium (Pinsky, 2020). Our observations on diastolic performance are of great importance in, for example, experimental studies on heart failure with preserved ejection fraction.

### 4.3 Ventilatory parameters

The lower CO and slow peripheral perfusion allowed a longer time for oxygen extraction in the tissues, confirmed by an increased arteriovenous oxygen difference. The returning mixed venous blood had higher levels of metabolites such as CO_2_ and lower levels of oxygen. We did not find other studies that have compared ventilatory parameters before and after sternotomy.

### 4.4 Limitations

The study has some limitations to consider. First, the sample size was modest, causing several statistically near‐significant results, whereas the magnitude of estimated cardiopulmonary changes is scientifically and clinically meaningful and should also be considered in the interpretation of the present results. Second, the study was performed on pigs and might not be similar in other animal models. Especially, we acknowledge that the closed‐chest approach in rodents or other small animals has some technical difficulties that might not be possible to overcome. Third, propofol is a known cardiovascular depressant. However, our anaesthetic approach is widely used and the results are externally valid. The use of ketamine or volatile anaesthetics might cause different results. Lastly, the study was performed on animals that were enrolled in a study on long‐term pulmonary embolic disease. The baseline values were generally within normal limits, but with few alterations. Accordingly, the results might not be representative for all porcine cardiovascular models.

## 5 CONCLUSIONS

We demonstrated and quantified cardiopulmonary haemodynamic differences between open‐ and closed‐chest approaches. Key haemodynamic variables were significantly affected. Given that no consensus or guidelines exist, researchers doing invasive phenotyping should consider an instrumentation approach most closely related to the imitative cardiovascular disease to ensure rigour and reproducibility in preclinical research.

## AUTHOR CONTRIBUTIONS

The experiments were performed at the Department of Clinical Medicine, Aarhus University. Conception and design of the work: Mathilde Emilie Kirk, Niels Moeslund and Mads Dam Lyhne All authors contributed to acquisition, analysis or interpretation of data and to drafting of the work or revising it critically for important intellectual content. All authors have approved the final version of the manuscript and agree to be accountable for all aspects of the work in ensuring that questions related to the accuracy or integrity of any part of the work are appropriately investigated and resolved. All persons designated as authors qualify for authorship, and all those who qualify for authorship are listed.

## CONFLICT OF INTEREST

None declared.

## Supporting information



Statistical Summary Document

## Data Availability

The data that support the findings of this study are openly available in figshare at: https://figshare.com/s/f3e4029b31e47ac02626 (Supporting Information Table S1) and https://figshare.com/s/ca1e9fc5914833bf1c59 (Supporting Information Table S2).
